# Point‐of‐Care Ultrasound M‐Mode Analysis Using *M.mode.ify*: An External Validation Study for E‐point Septal Separation Measurement

**DOI:** 10.1002/jum.70349

**Published:** 2026-06-24

**Authors:** David Crockett, Elijah Wyatt, Benjamin C Smith, Jamal Jones, Jennifer Cotton, Christopher Kelly

**Affiliations:** ^1^ Department of Emergency Medicine University of Utah Salt Lake City Utah USA; ^2^ Department of Emergency Medicine University of Tennessee College of Medicine Chattanooga Tennessee USA

**Keywords:** EPSS, M‐mode, point‐of‐care ultrasound

## Abstract

**Objectives:**

M‐mode ultrasound provides high temporal resolution imaging by rendering a single scan line over time, enabling precise measurement of rapidly moving structures. Traditional M‐mode is limited to real‐time acquisition and cannot be applied retrospectively. *M.mode.ify* is a novel software tool that generates M‐mode‐like tracings from video data, enabling post‐acquisition analysis. This study evaluates the accuracy of *M.mode.ify* in measuring E‐point septal separation (EPSS), a key echocardiographic parameter.

**Methods:**

We retrospectively analyzed 50 cardiac ultrasound studies from our institution's quality assurance database. Inclusion criteria required both a saved M‐mode EPSS image and a parasternal long axis (PLAX) video clip. EPSS values from M‐mode served as the reference standard. Two blinded reviewers independently measured EPSS using *M.mode.ify*. Inter‐rater reliability and agreement with the standard were assessed using Pearson correlation coefficients. Absolute error was calculated to quantify measurement precision.

**Results:**

Of the 50 analyzed studies, 9 were excluded due to inadequate anatomical visualization. Among the 41 included studies, inter‐rater correlation was strong (*r* = 0.90), and agreement between *M.mode.ify* and standard M‐mode measurements was excellent (r = 0.92). The mean absolute error was 2.38 mm (SD 1.71 mm).

**Conclusion:**

*M.mode.ify* demonstrates high accuracy and reliability in measuring EPSS retrospectively. This tool may expand the utility of ultrasound data by enabling post hoc analysis of M‐mode parameters.

**Abbreviations**
EPSS: E‐point septal separation
LVEF: left ventricular ejection fraction
LVFS: left ventricular fractional shortening
PLAX: parasternal long axis
POCUS: point‐of‐care ultrasound
TAPSE: tricuspid annular plane systolic excursion

M‐mode ultrasonography is an important component of cardiac imaging, offering temporal resolution by capturing motion along a single scan line at a sampling frequency exceeding 1000 Hz.^1^, ^2^, ^3^, ^4^ This capability allows for evaluating dynamic cardiac structures, such as cardiac valve motion, and for calculating parameters like E‐point septal separation (EPSS), which correlates with left ventricular function.^3^, ^5^, ^6^, ^7^ Despite its utility, M‐mode is constrained by its real‐time nature; measurements must be made during the scan, limiting retrospective assessment. To address this limitation, *M.mode.ify*, Smith et al developed a software tool that reconstructs M‐mode‐like images from standard ultrasound video clips.^8^ The frame rate of video data (typically 29–52 Hz) is significantly lower than that of true M‐mode, raising concerns about measurement accuracy.^9^ To date, several studies have utilized *M.mode.ify*, but to our knowledge there has not been an external validation.^10^, ^11^ This study aims to externally validate the use of *M.mode.ify* for retrospective point‐of‐care ultrasound (POCUS) M‐mode EPSS measurements by comparing its output to standard M‐mode tracings.

## Material and Methods

### Study Design

We conducted a retrospective observational study using POCUS studies performed in the emergency department at the University of Utah.

### Data Collection

Our dataset was obtained from POCUSs stored in our quality assurance database. These ultrasound studies were captured by a wide range of training levels including attending physicians, ultrasound fellows, and emergency medicine residents. The ultrasounds were performed on GE venue GO, Sonosite, and Mindray machines. We extracted the 50 most recent studies performed prior to our study design from the institution's quality assurance database. Inclusion criteria included a saved M‐mode image with a documented EPSS measurement and a corresponding video of the parasternal long axis (PLAX) to be utilized for *M.mode.ify* validation.

### Ground Truth Protocol

The ground truth EPSS measurement was extracted from the value recorded at the bedside by the sonographer and stored as an image with the ultrasound study (Figure 1A). The sonographer's training ranged from third‐year medical student to ultrasound fellowship‐trained and boarded emergency medicine attendings. We treated this data as correct and it was reviewed and validated by ultrasound fellowship‐trained physicians.

**Figure 1:**
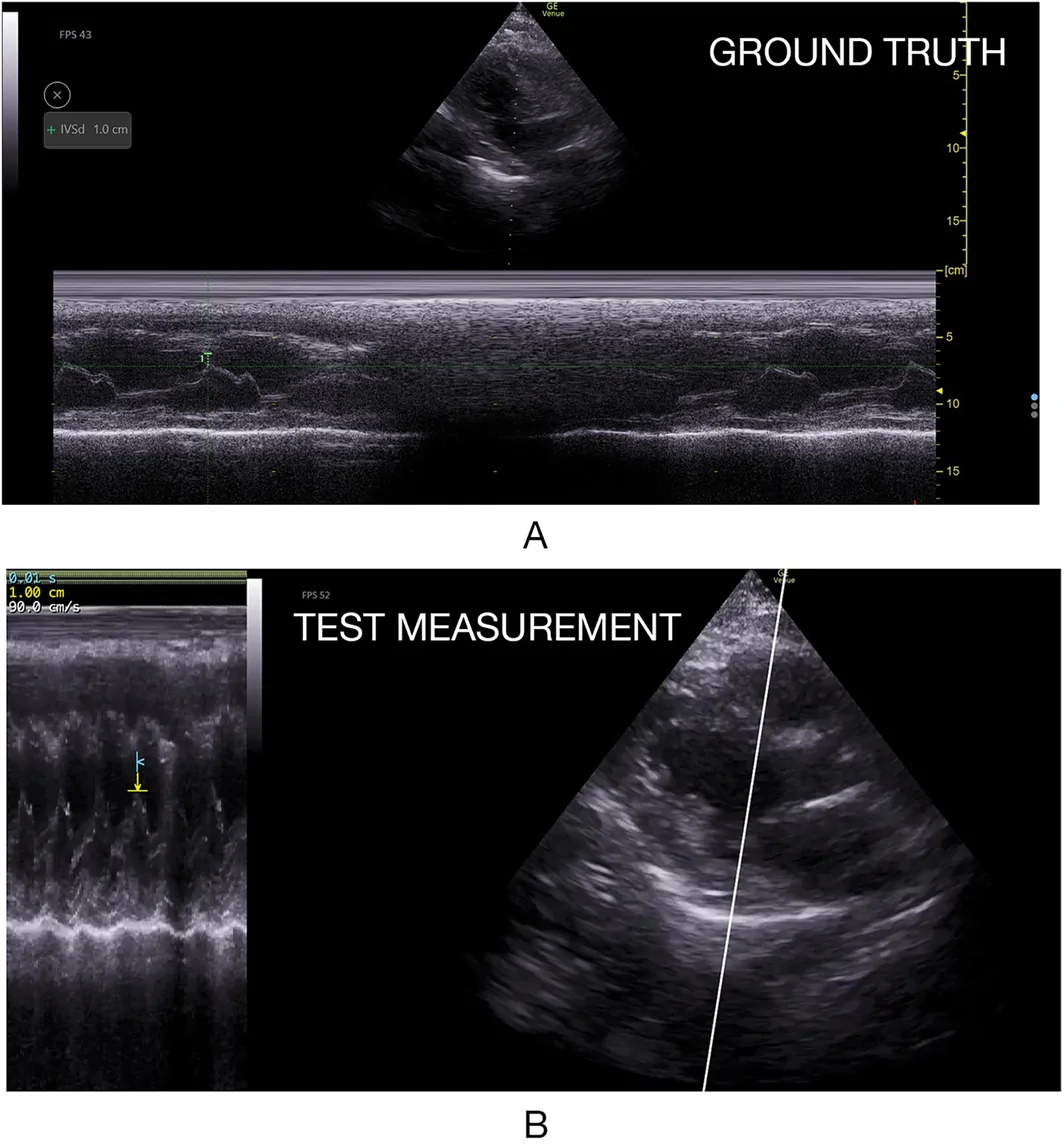
**A**, Ground truth image obtained by m‐mode measurement with ultrasound machine. **B**, Test measurement computed with *M.mode.ify* from video file saved with the ground truth ultrasound study.

### Test Measurement Protocol

The test EPSS measurement was performed by 2 authors (EW, BS) at a separate institution from the utilized POCUS clips. Both authors are ultrasound fellowship‐trained attending physicians, and the *M.mode.ify* software was programmed by BS.

Each author measured the EPSS value from a PLAX video with *M.mode.ify* and then selected the video frame with maximal anterior mitral valve motion and measured the distance to the septum. The final test EPSS value was the average of the 2 reviewers' measurements. If the reviewer could not find a video frame with a clear view of the anterior mitral valve and septum, that study was excluded from analysis. Both reviewers had to agree on quality for the study to be included. The reviewers were blinded to the ground truth measurements. An example usage of the *M.mode.ify* tool is shown in Figure 1B.

### Statistical Analysis

We assessed inter‐rater reliability and agreement between test and ground‐truth measurements using Pearson correlation coefficients (*r*). The absolute error between test and reference values was calculated to evaluate precision. Statistical analysis was performed using the Pandas scientific library for the Python programming language.^12^

## Results

Of the 50 studies reviewed, 9 were excluded due to inadequate visualization of cardiac anatomy. The remaining 41 studies were included in the final analysis. Inter‐rater reliability (*r*‐value) was found to be 0.90. Agreement (*r*‐value) with reference M‐mode measurement was found to be 0.92 with a mean absolute error of 2.38 mm (SD 1.71 mm, 95% CI: 1.84–2.92 mm) (Figure 2). These results indicate strong agreement between *M.mode.ify*‐derived measurements and traditional M‐mode measurements with minimal variability.

**Figure 2:**
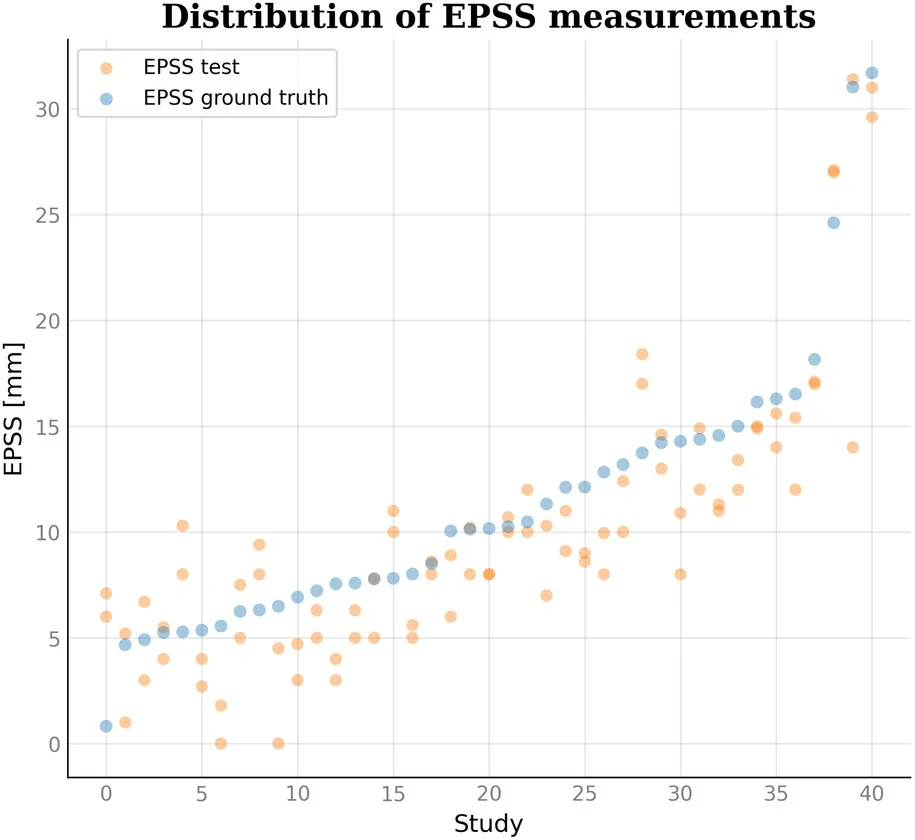
Distribution of EPSS ground truth and test measurements plotted in ascending order.

## Discussion

EPSS is a quantitative echocardiographic measurement used to estimate left ventricular ejection fraction (LVEF), a key indicator of systolic function.^7^, ^13^, ^14^, ^15^ EPSS is obtained from the PLAX view using M‐mode, by measuring the distance between the anterior mitral valve leaflet at its maximal opening during early diastole and the interventricular septum. An EPSS greater than 7 mm is generally associated with reduced LVEF, and values above 13 mm often indicate severely impaired systolic function (LVEF ≤35%).^14^, ^15^

Our findings support the use of *M.mode.ify* as a reliable tool for retrospective EPSS measurement on POCUS videos. Despite the lower frame rate of video data, the software achieved excellent correlation with standard M‐mode measurement and demonstrated high inter‐rater reliability. This capability is particularly valuable in clinical and educational settings where real‐time M‐mode acquisition may be inadvertently missed or unavailable. *M.mode.ify* enables clinicians to extract meaningful quantitative data from archived ultrasound videos, potentially improving diagnostic accuracy and workflow efficiency.

Despite our high correlation, there are some limitations users of *M.mode.ify* should be cognizant of. Given we found an absolute mean error of 2.38 mm, we recommend caution in values around normal/abnormal cutoffs as this error difference may make a measurement fall on either side of a dichotomous value cutoff. Significantly abnormal or obviously normal values would be largely unaffected by this small difference and therefore can be more reliably accepted. Additionally, because *M.mode.ify* allows users to select the specific cardiac cycle from which to measure EPSS, there is potential for beat‐to‐beat variability. This user‐dependent selection may contribute to slight differences in measurements, such as those in patients with arrhythmias, conduction delays, valvular pathologies, among others. Additionally, the resolution of *M.mode.ify* decreases with an increased heart rate and special caution should be given to measurements in patients with tachycardia.

This limitation in resolution may have contributed to the 9 studies that were excluded due to inadequate visualization of cardiac anatomy. In these excluded studies the sonographer saved an m‐mode image that allowed measurement of EPSS, however the subsequent video clips stored with the study did not delineate adequate anatomy in *M.mode.ify*. This maybe be due to inferior video quality, or, to the increased temporal resolution of m‐mode that overcomes limitations in quality.

The ability to extract quantitative data from archived ultrasound videos has significant implications for clinical practice, education, and research. In environments with high utilization of POCUS where rapid decision‐making is essential and image acquisition conditions may vary, *M.mode.ify* offers a valuable opportunity to re‐analyze cardiac function metrics post hoc. This can enhance diagnostic confidence, support quality assurance efforts, and facilitate training by allowing learners to review and measure key parameters after the initial scan.

EPSS was chosen as the measurement for this validation of *M.mode.ify* as it was a measurement that has not been previously reported in the literature (as compared to tricuspid annular plane systolic excursion [TAPSE] and left ventricular fractional shortening [LVFS]).^10^, ^11^ Additionally, EPSS is a commonly obtained bedside measurement which allowed for adequate data collection with regards to the number of available studies. The utilization of EPSS as a measurement should be cautioned as data on its accuracy is lacking.^16^ The use of EPSS in this study should not be viewed as an endorsement of the measurement but rather was a convenient way to validate the *M.mode.ify* tool.

Future studies may explore the application of *M.mode.ify* to other M‐mode‐dependent measurements and assess its performance across diverse patient populations and clinical scenarios. As ultrasound technology continues to evolve, tools like *M.mode.ify* that extend the utility of existing data will play an important role in maximizing the value of POCUS.

*M.mode.ify* can be accessed at: https://coreultrasound.com/m-mode-ify/.

## Conclusion

This study demonstrates the reliability and accuracy of *M.mode.ify* for measuring EPSS from POCUS video clips. These findings suggest that *M.mode.ify* can serve as a practical tool for retrospective EPSS analysis.

## Data Availability

The data that support the findings of this study are available from the corresponding author upon reasonable request.
